# Torsion-Mediated Interaction between Adjacent Genes

**DOI:** 10.1371/journal.pcbi.1003785

**Published:** 2014-09-04

**Authors:** Sam Meyer, Guillaume Beslon

**Affiliations:** Université de Lyon, INSA Lyon, INRIA, LIRIS, CNRS UMR5205, Lyon, France; Rutgers University, United States of America

## Abstract

DNA torsional stress is generated by virtually all biomolecular processes involving the double helix, in particular transcription where a significant level of stress propagates over several kilobases. If another promoter is located in this range, this stress may strongly modify its opening properties, and hence facilitate or hinder its transcription. This mechanism implies that transcribed genes distant of a few kilobases are not independent, but coupled by torsional stress, an effect for which we propose the first quantitative and systematic model. In contrast to previously proposed mechanisms of transcriptional interference, the suggested coupling is not mediated by the transcription machineries, but results from the universal mechanical features of the double-helix. The model shows that the effect likely affects prokaryotes as well as eukaryotes, but with different consequences owing to their different basal levels of torsion. It also depends crucially on the relative orientation of the genes, enhancing the expression of eukaryotic divergent pairs while reducing that of prokaryotic convergent ones. To test the *in vivo* influence of the torsional coupling, we analyze the expression of isolated gene pairs in the *Drosophila melanogaster* genome. Their orientation and distance dependence is fully consistent with the model, suggesting that torsional gene coupling may constitute a widespread mechanism of (co)regulation in eukaryotes.

## Author Summary

During the transcription process, the genetic sequence encoded in the DNA molecule is expressed by an enzymatic complex. This process is often considered as independent for each gene, despite numerous reported cases of one transcribed gene perturbing a neighbor gene's expression, which is then regarded as a side-effect. Here, we suggest in the contrary that such interactions are a widespread feature, resulting from the propagation along the DNA molecule of mechanical stress generated during gene transcription. This torsional stress modifies the facility with which the transcription machinery separates the two strands of the double-helix in order to access the bases, and thus the expression level of any gene located nearby. We develop a quantitative model of this effect, showing that it depends strongly on the orientation of the genes, which is confirmed by the analysis of *in vivo* expression levels in the drosophila genome. This observation suggests that torsional coupling may play an important role in genetic regulation, and might favor the orientation-dependent co-localization of genes involved in similar functions, which need to be expressed together.

## Introduction

Transcription involves the separation of the two DNA strands by the RNA Polymerase complex during the initiation phase. The formation of this “transcription bubble” [1] can represent a significant energetic cost, determined by the universal thermodynamic properties of the DNA molecule, and may thus constitute a widespread mechanism of gene regulation. This cost depends strongly on the promoter sequences, which are usually thermodynamically unstable [2], [3]. But it also depends crucially on the presence of torsional stress [4] giving rise to supercoiling, a mechanical feature present in virtually all biological transactions involving DNA [5], [6], and in particular transcription and replication. A negative torsion results in a negative superhelical density, quoted 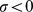, and destabilizes the double helix, facilitating the spontaneous formation of transient denaturation bubbles even at low temperature. Conversely, the double-helical state is stabilized by a positive torsion [2]. This mechanism is widely relevant to prokaryotic regulation, with most bacteria having a globally underwound genome allowing the spontaneous opening of promoters, while many “thermophilic” organisms constrain this torsional stress to a positive level, which could thus be one of the mechanisms ensuring the stability of the double-helix even beyond the usual melting temperature [7], [8]. In eukaryotes, free DNA was found to be torsionally unconstrained at the global scale, and the role of supercoiling was often neglected for this reason. However, recent experiments demonstrated the presence of important levels of supercoiling in local “topological” domains [9], [10], which probably play a functional role.

**Figure 1:**
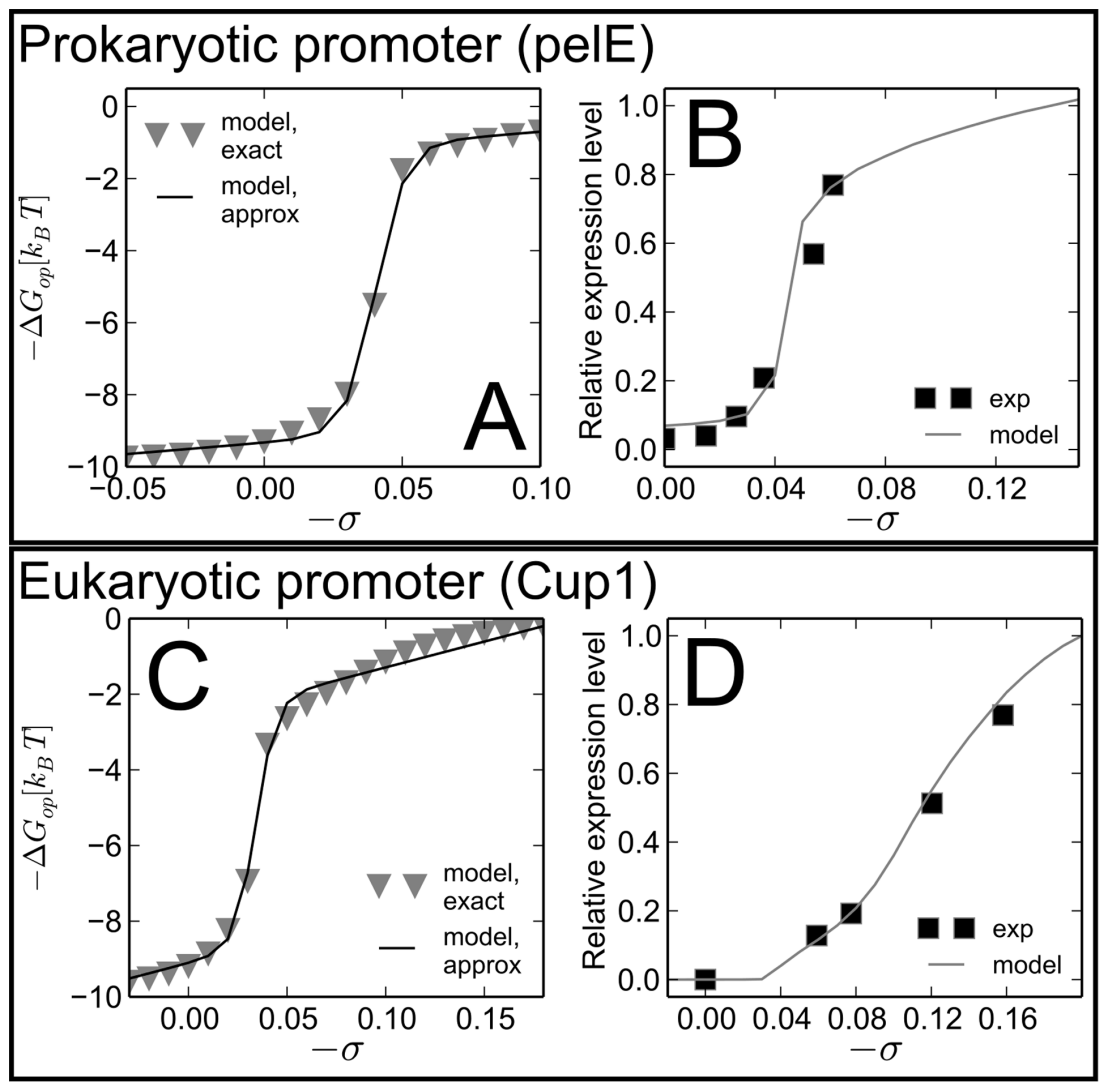
Supercoiling-dependent opening profile (A–C) and expression level (B–D) of the *pelE* bacterial promoter (upper panel) and the *CUP1* yeast promoter (lower panel). Experimental datapoints and sequences are taken from [11] and [12] respectively: in each case, the gene was inserted on a plasmid and transcribed with either bacterial RNA polymerase (B) or the eukaryotic polymerase II (D). Note that the dependence was tested up to a much higher level of supercoiling in (D). The opening profiles (left) are computed without any free parameter (triangles), and fitted with a sigmoidal curve (solid line) for the subsequent simulations. The experiment with *pelE* included the regulatory protein Crp (see Models) and the calculation of the opening profile included the 60 bases ahead of the transcription start site. The *CUP1* experiment included no transcription factor, but included a 410 bp sequence ahead of the promoter, which was also included in the calculation. The transcription models involved effective temperatures of 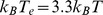 and 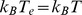 respectively (see Models).

*In vitro* experiments have shown the influence of supercoiling in both prokaryotic and eukaryotic transcription, as shown in Fig. 1. The bacterial promoter of *pelE*, inserted on a plasmid, is expressed by bacterial polymerase only when the DNA is underwound at a level similar to the *in vivo* average level of −0.06 (B) [11]. Eukaryotic RNA polymerase II, in contrast, is able to transcribe the yeast *CUP1* promoter on a torsionally relaxed plasmid, but only in the presence of a minimal set of *in vivo* relevant transcription factors, and in particular TFIIH which contains an ATP-consuming helicase subunit ensuring the formation of the transcription bubble [12], [13]. But remarkably, when the plasmid is negatively supercoiled, the gene can be transcribed by RNA PolII *in absence of any transcription factor* [12], in which case the expression level increases with the applied torsional stress (Fig. 1D). While this mode of regulation is probably not dominant *in vivo*, it could very well play a role for those genes located in underwound domains. Interestingly, for these two very different systems, the expression rate is proportional to σ^2^, *i.e.* precisely the expected dependence of the promoter opening free energy, arising from the elastic cost of unwinding double-helical DNA [12] (Fig. S1). Altogether, accumulating data come in support of the long-proposed idea [4] that supercoiling-dependent promoter opening could be an important regulator of transcription, not only in prokaryotes [11], [14], [15] but also (and differently) in eukaryotes [3], [12], [16].

Conversely, an important source of supercoiling *in vivo* is transcription itself [10], [17]. In the elongation phase where the RNA Polymerase complex advances along the gene sequence, it has to turn around the DNA axis following the helical geometry of the molecule. In 1987, Liu and Wang [18] postulated that the frictional drag of this large complex would impede such a rotational movement; rather, the DNA strands would be twisted, resulting in a considerable accumulation of positive superhelical stress ahead of the transcription machinery, and negative behind it. This important stress originates from the transcription unit, and propagates along the DNA molecule over a few kilobases [19]–[22], where it is progressively released by specific enzymes (topoisomerases, gyrases) existing in all organisms [22], [23]. *In vivo* measurements suggested that this transcription-induced supercoiling is probably a major determinant of “topological domains” in eukaryotic as well as bacterial chromatin [9], [10], [17].

These two reciprocal aspects of transcription-supercoiling coupling have been known for decades. Their *combination* immediately suggests that the transcription of adjacent genes could be coupled by the propagation of torsional stress along the DNA. This mechanism has already been suggested and experimentally demonstrated in specific examples of both prokaryotic [14], [24] and eukaryotic [21], [25], [26] divergent promoters. Moreover, genome-wide analyses of sequence motifs associated to torsionally-induced DNA structural transitions have illustrated the possible widespread role of torsion in the regulation of nearby promoters in bacteria [15], [27]. However, there is no systematic and quantitative description on how nearby genes could mutually affect their expression through supercoiling, and how this coupling would then depend for example on the relative orientation and distance of the genes. Since it relies on physical properties of DNA, this effect is likely to universally affect eukaryotic and prokaryotic organisms, although with different effects owing to the different level of supercoiling in these organisms or to their different gene densities. In eukaryotes in particular, some studies suggest that DNA supercoiling could account for co-regulation of neighbor genes [26].

In this paper, we propose a simple theoretical framework for this interaction, which allows exploring the role of different parameters (promoter orientation and distance, gene length, basal superhelical density…) on the time-averaged co-expression of neighbor genes. The model focuses on the most generic features of the interaction since they prevail at the genome-wide level. It voluntarily leaves aside several specific aspects of promoters response to supercoiling. The proposed mean-field description is derived from the knowledge-based physical properties of the double-helix, and requires only few adjustable parameters to quantitatively reproduce the behavior of model experimental systems in very different (prokaryotic or eukaryotic) organisms. By extrapolating this behavior to different parameters, it allows to predict the differential effect of the torsional coupling in a broad range of conditions and organisms.

Interestingly, despite the very different *global* role of supercoiling in prokaryotic and eukaryotic regulation, the *local* torsional perturbation is predicted to affect the regulation of nearby genes in both types of organisms (albeit differently), in particular in the case of symmetrically oriented (divergent or convergent) genes, as already observed [24]–[26], [28]. This perturbation differs substantially from usually proposed mechanisms of “transcriptional interference”, which assume that the adjacent genes overlap, or experience a collision or sharing of their transcriptional machineries [28], [29]. Here, we suggest that *even without any molecular contact* between the machineries expressing distant genes, the propagation of torsional stress along the DNA could significantly couple (positively or negatively) their expression. Finally, we show that the predictions of the model are supported by published expression data of *Drosophila melanogaster*, where the expression of isolated gene pairs significantly depends on their orientation and distance.

## Results

### Reciprocal interplay between transcription and supercoiling

The destabilization of the double-helix by torsional stress is a well-known phenomenon [4], which was shown to play an important role in the global regulation of both prokaryotic [15] and eukaryotic [30] promoters. As an example, it is involved in the rapid response of bacteria to an external stress, where all promoters must rapidly modify their expression in a coordinated manner [11], [14]. This mechanism has been quantitatively described using at least two different physical models of DNA, that of Benham and coworkers [2], [27] and the mesoscopic Peyrard-Bishop-Dauxois model [3], [16]. Both models are based on measured thermodynamic and elastic properties of the base-pairs [2], [8], and estimate the supercoiling-dependent opening free energy of the double-helix. Here, we use a recent efficient implementation of the former model [31], [32], and integrate it into a thermodynamic model of transcription [33], [34], which then allows to compute the average transcription rate of a promoter of given sequence (Fig. 1). The melting profile predicted by the DNA model typically exhibits a sharp transition around 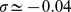 (Fig. 1A), with the opening probability increasing with the applied negative supercoiling.

The proposed framework is based on the hypothesis that the transcription level is proportional to the initiation probability, as estimated from the chemical equilibrium between the bound and unbound states of the transcription machinery. We note however that the formation of the transcription bubble is not a purely thermal process, but is rather facilitated by conformational changes in the RNA polymerase complex, which may depend on the type of polymerase of the organism (in particular the bacterial polymerase vs. the energy-consuming eukaryotic Polymerase II) [1]. This non-thermal energy scale is taken into account by introducing an effective temperature, which is then the only adjustable parameter of the transcription model and can be calibrated on *in vitro* experimental data (see Fig. 1 and Models section). This description neglects a part of the promoter specificity in the initiation stage, and other regulation mechanisms in the subsequent stages of transcription (see Models). Despite these simplifications, the model quantitatively reproduces the expression profiles of the model systems (Fig. 1). In these experiments, the superhelical level is fixed by the number of superhelical turns imposed in the plasmids where the gene is inserted. In the following of the study, we extrapolate this response curve to promoters located on the chromosome(s), where the external source of supercoiling is different, and where the model then allows to make predictions for a broad range of situations without any additional parameters. This simplicity is a key advantage for our model focusing on the most generic consequences of the torsional coupling between adjacent genes at the genome-wide scale. The reader should however keep in mind that more specific features are not taken into account, in particular the subtle competition between different stress-induced transitions [15] which are known to affect the opening rates of bacterial promoters, and allow for a fine tuning of the supercoiling-dependent regulation with the help of DNA-binding proteins [14] (see Discussion). In the following we focus on the simpler situation where the opening of the initiation site is the only structural transition absorbing the superhelical stress.

The superhelical stress involved in transcriptional regulation can have different origins. In prokaryotes, this level is controlled at the global scale by ATP-consuming enzymes [7], [14]. In eukaryotes, the situation is very different, since nucleosomes cover most of the genomic DNA and store a constrained level of supercoiling [6], while free DNA is torsionally relaxed in average. However, both types of organisms exhibit local variations of these values in so-called topological domains [9], [10], [17], which could be generated by transcription. While previous studies have focused on the promoter response to a fixed level of supercoiling, in this paper we consider the specific case where the external source of supercoiling is the transcription of a nearby gene along the DNA molecule, and we quantify how its influence then depends on the distance, length and orientation of the genes.

**Figure 2:**
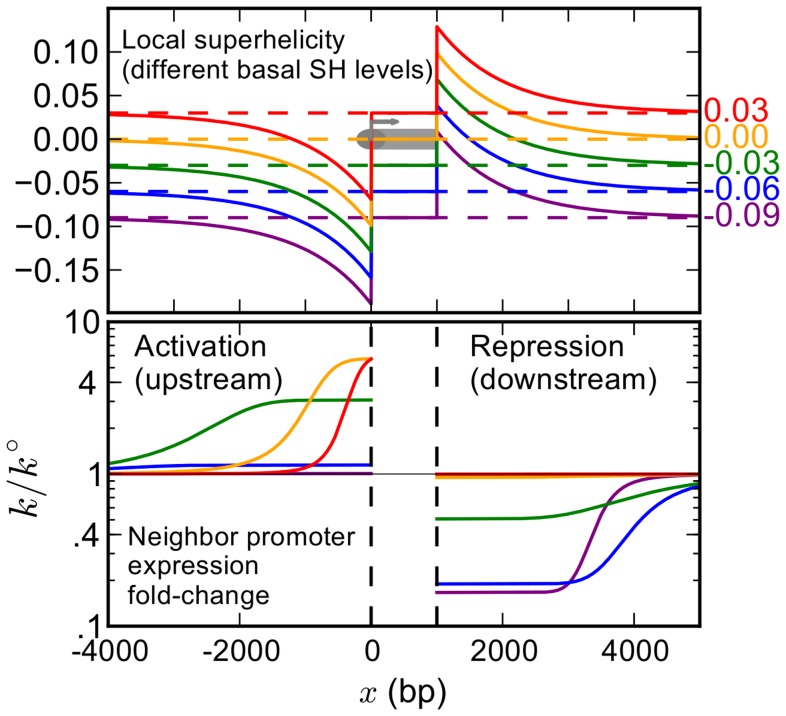
Illustration of the effect of a transcribed gene on the local distribution of superhelical density (upper panel) and resulting expression fold-change of a nearby promoter (lower panel), for different values of the basal superhelical density. In prokaryotes, the average superhelical density can vary in the range 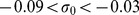, depending on the organism, the stage in the growth cycle [14], and the genomic site, with an average level 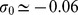 for *E. Coli*. In eukaryotes, the average superhelical density of *free* DNA is 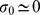, but this level can vary along the genome [9], [10] and is strongly affected by the presence of nucleosomes. The active gene is indicated as a gray box, and for simplicity we assume a common gene length (1 kb) and transcription level for all values of σ_0_, and the sequence of the CMV promoter (opening profile in Fig. S2). See details of the employed parameters in the Models section.

The transcribing polymerase acts as a torsional motor that generates positive superhelical stress ahead of the complex, and negative stress behind it [18], [35]. This stress propagates along the DNA double-helix [10], [21], and is progressively released by specific enzymes (topoisomerases), but also, in the case of eukaryotes, by the release of nucleosomes [22]. In this paper, we neglect the dynamic aspects of the process and consider its time-averaged approximation consistent with the thermodynamic model of transcription, which can then be described using a mean-field approach. Assuming that the stress is progressively released outside the gene with uniform efficiency, the resulting time-averaged distribution of superhelical stress decays exponentially from the transcription unit (see Models and Fig. 2, upper panel, with different basal levels of supercoiling).

This profile is consistent with various measurements obtained *in vivo* with different protocols, involving either the intercalation of a psolaren-based agent in underwound DNA [21], structural transitions of the double-helix [19] or a supercoiling-sensitive promoter [20]. While the properties of this propagation could be expected to depend on the considered system (topoisomerase concentration, DNA sequence…), these very different *in vivo* experiments reported remarkably consistent propagation distances of around 1000 bases. Surprisingly, this value was observed not only in prokaryotic, but also in eukaryotic organisms, suggesting that nucleosomes do not modify significantly the propagation distance (see Discussion). We note however than only the psolaren-based experiment [21] was calibrated so as to provide a direct and quantitative measure of the level of superhelical density; other methods were either more indirect [24] or provide only a qualitative estimate of the supercoiling level associated to the employed probe of supercoiling [19], [20]. Future experiments might therefore allow refining these estimates, and distinguishing the propagation modes in different organisms. In this paper, based on the available experiments, we use the value of 1000 bp for the propagation distance as a parameter in the model, for both prokaryotes and eukaryotes. The amplitude of the perturbation is assumed proportional to the transcript length and the promoter strength, consistent with the idea that the torsional stress accumulates as the polymerase unwinds the two DNA strands along the gene (one turn every 10 base-pairs) and confirmed by experimental data [24]. The parameters of the models are adjusted so that the generated levels of supercoiling are compatible with the data of Kouzine et al. [21].

### Interaction between adjacent genes

Fig. 2 shows the local distribution of supercoiling, as obtained from the previously described model of transcription for an illustrative gene of 1 kb. The different displayed curves correspond to illustrative basal levels typical of prokaryotes in different growth phases, from −0.03 in the ATP-poor stationary phase to −0.09 in specific cases of external shock [14]. Higher levels are rather relevant to eukaryotes, where free DNA is torsionally unconstrained in average (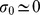), but can also vary along the genome [9], [10], with possible causes including transcription and other dynamic processes involving nucleosomes. For simplicity, we used a common gene length of 1 kb in all cases, which is illustrative of many prokaryotic as well as eukaryotic genes. Note that in eukaryotes, the average gene length is often larger (5 kb in *Drosophila melanogaster*, and 10–20 kb in mammals), but this number is strongly affected by a minority of very long genes (up to 2 Mb for humans). In contrast, the median length, which reflects the majority of the genes, is closer to 1 kb (*e.g.* 1.75 kb for *D. melanogaster*). Our illustrations are therefore relevant to most eukaryotic genes, but not to very long genes where the elongation kinetics probably plays an important role.

If another promoter is located within a few kilobases of the transcribed gene, the curves of Fig. 1 suggest that the locally generated superhelical stress may modify its opening properties, and thus its transcription level. Actually, one of the methods used to monitor the transcription-induced supercoiling is based precisely on this property, in which case the torsional response of the employed probe promoter must first be calibrated [20], [24]. By combining these distributions with the supercoiling-dependent transcription rate as described in the previous paragraph, we are able to predict the modification of transcription rate due to the transcriptional interaction (Fig. 2, lower panel). Unsurprisingly, the transcription is reduced when the promoter is located downstream of the transcribed gene, and increased when upstream; this effect decreases with distance in a non-trivial way due to the nonlinear opening profile of the promoter. With this mechanism depending only on the universal physical properties of the double-helix, it is likely to affect all types of known organisms. However, and importantly, because of the different basal levels of prokaryotes and eukaryotes, the predicted effects are different. In bacteria, the promoters are mostly “open”, and the repressive effect tends to be stronger than the inductive one. In eukaryotes conversely, negative stress generated locally by transcription could significantly increase the expression level of any gene located upstream of the promoter.

**Figure 3:**
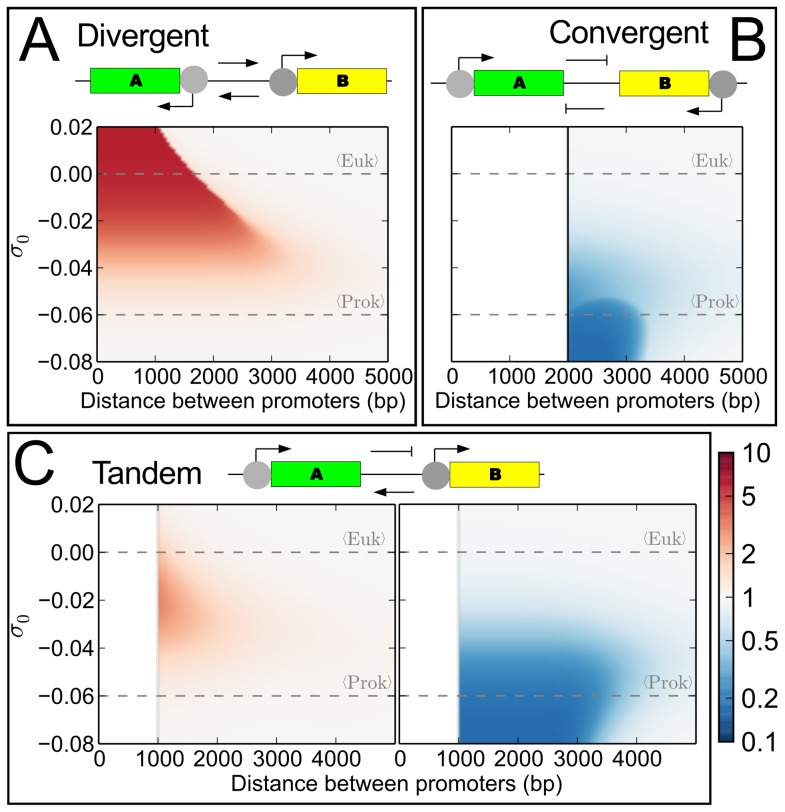
Expression level fold-change resulting from the dynamic coupling between neighbor genes in (**A**) divergent, (**B**) convergent, or (**C**) tandem arrangements, as a function of promoter distance and basal superhelical level. In the first two cases, the construction is symmetric and only one gene is shown. Dashed gray lines indicate the average superhelical levels in eukaryotes and prokaryotes. Note that these levels can exhibit significant local and temporal variations [9], [10], [14], as described in the caption of Fig. 2. The parameters used in the simulation are described in Models; for simplicity, the two genes are 1000 basepairs long and controlled by identical CMV promoters (opening profile in Fig. S2).

Fig. 2 shows only the effect of one transcribed gene on the neighbor's promoter. However, we expect that the second gene will in turn also influence the former's promoter, and modify its expression. The level of each promoter is therefore the result of a dynamic equilibrium between the two genes. Applying the model developed in the previous section, this level can be determined numerically with a simple algorithm. Starting with the whole stretch of DNA at the basal supercoiling level of the considered region/organism, we iteratively compute the expression level of each promoter, and adjust the supercoiling profile accordingly until reaching a fixed point. Unsurprisingly, the effect of the interaction depends crucially on the relative *orientation* of the two genes, as shown on Fig. 3. The figure shows that its strength is also a function of the distance between the promoters and the basal superhelical density of the organism (dashed lines indicate the average value of this density for prokaryotes and for free DNA in eukaryotes).

In the case of *divergent* promoters (A), each promoter favors the expression of the neighbor, which in turn increases the former's activity. The diagram of the dynamic system shows that the effect is predicted to be stronger for eukaryotic organisms, and decays sharply at a cutoff distance that decreases with the basal superhelical level. Note that the diagram shows the *relative* change in expression level; in general, for a given promoter sequence, the *absolute* basal level is higher for lower values of σ_0_ (Fig. 1). In contrast, *convergent* promoters are mutually repressive (B). This type of interaction in well-known in biochemical networks, and can lead either to a global reduction of both expression levels, or to the selective extinction of one of the two genes. This repressive effect is predicted to affect bacteria more strongly than eukaryotic organisms. Finally, for genes in *tandem* (C), the interaction is more subtle, with an asymmetrical influence leading to a limited increase of the upstream gene and repression of the downstream gene, especially at intermediate basal levels of supercoiling where both effects may coexist.

The presence of an interaction between the transcription of neighbor genes is often referred to as “transcriptional interference” in the biological literature, and has been reported in many studies [28], [29]. We noted that supercoiling has already been evoked as a possible mechanism for divergent promoters, but only in a few studies that specifically address this topic [24]–[26]. In contrast, most general papers on transcriptional interference assume a direct molecular contact between the transcription machinery of the genes, either by collision if the genes or their promoters overlap, or by incorrect termination (read-through), or simply if they share the same individual regulatory protein or polymerase [36]. It is interesting to note that the torsional coupling proposed here implies that any two genes distant of less than ∼3000 bases experience a mutual influence *without any interaction of their transcription machineries*, simply by propagation of the DNA mechanical deformations.

### Torsionally coupled gene pairs in the *Drosophila melanogaster* genome

The proposed torsional coupling implies that neighbor genes are not independent, but coupled in an orientation-dependent way. Genome-wide expression analysis studies have demonstrated the co-expression of adjacent genes in yeast [37] as well as plants [38] and mammals [39]. Among these coexpressed pairs, *divergent* genes were found to be the most frequent as well as more expressed and highly correlated [37], [38], [40], [41], and also less noisy [41], while convergent gene pairs were under-represented [29]. A fraction of these divergent pairs are “bi-promoters”, where a single bidirectional promoter controls the two genes of the pair, in which case a transcriptional coupling can indeed be expected without any torsional effect. For the majority of the genes where the promoters are separate, the proposed explanation for the co-expression is that neighbor genes may often belong to the same chromatin domain, with similar expression properties, as identified by biochemical marks [42]. But while the chromatin state does certainly play a crucial role in these correlations, we do not expect this effect to depend on the pair orientations. Several authors argue that divergent promoters are often closer, and the effect should thus be stronger in this case than for tandem and convergent promoters. But the only identified lengthscales associated to chromatin regulation are either larger, with topological and epigenetic domains of 10–1000 kb [9], [42], or smaller, with a co-regulation being expected if the two promoters belong to the same nucleosome (200 bases). Between these two scales, it is difficult to predict how the correlations should depend on the distance; this dependence could even be non-monotonous if genomic sites located 5 or 10 nucleosomes apart (1 or 2 kb) are spatially closest in the fiber and can share their transcription machinery. Moreover, if the different types of pairs are located randomly in the fiber, this effect would only explain a correlation, but not an *over-expression* of the divergent genes.

We suggest that the observed orientation-dependent expression features could be naturally explained by a torsional coupling between the genes. Interestingly, recent genome-wide measurements of supercoiling level showed that regions of gene clusters of several kilobases are subject to negative supercoiling correlated to the transcription level [9], [10]. A more detailed analysis of specific locations pointed to the particular effect of divergent genes, where the torsional coupling that we model here was directly observed [26]. To investigate the presence of such effects on a wider scale and in different orientations, we analyze the genome-wide expression of gene pairs from RNA-Seq expression data of 24 cell lines of *Drosophila melanogaster* [43]. We separate the torsional effects from other uncontrolled features, by focusing on “torsionally isolated” pairs of neighbors, *i.e.* pairs where (i) the genes are closeby, with the transcription units (start or end sites) less than 5 kb from the other gene's promoter and (ii) the two promoters are more than 3 kb away from any gene outside the pair, and therefore likely unperturbed by their transcription-induced torsion. This situation is rare in yeast where the genome is dense (and even more so in prokaryotes), and where short-range torsional interactions may form long chains of coupled genes, making it difficult to distinguish the proposed effect (see Discussion). In contrast, *D. melanogaster* has about 1400 of these pairs, representing nearly 20% of its genes. Among these pairs, 748 are divergent, 552 are in tandem, and only 103 are convergent. Note that these numbers do not necessarily indicate an evolutionary selection against convergent pairs: even with randomly distributed genes, our selection procedure eliminates more convergent pairs because their outwards promoters are more likely to be close to other genes.

**Figure 4:**
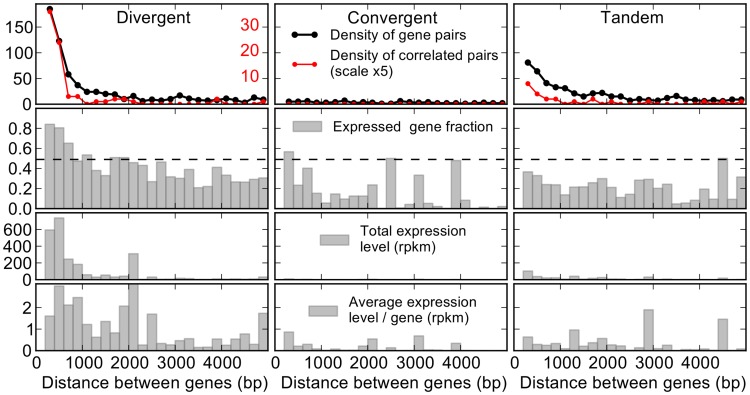
Expression patterns from torsionally isolated gene pairs of *Drosophila melanogaster*, *i.e.* the 1400 pairs of neighbor genes whose promoters are located at least 3 kb away from any other gene. The four rows indicate: (i) the total number of total (black) or correlated (red) pairs, in windows of 200 basepairs (mind the different scales), with a total number of 748 divergent, 552 tandem, and 103 convergent pairs. The same 200 basepair windows are employed in all rows. (ii) Fraction of expressed (nonzero transcript number) genes. The dashed line indicates the genome-wide average (around 0.5). (iii) Total and (iv) average transcription level, for the genes located in each 200-basepair window. Note that the profile of row (iii) simply reflects the product of row (i) and row (iv) (with a factor 2). Together, the two upper rows indicate that close divergent genes are (i) more frequent and (ii) more expressed than other genes in the considered sample. For tandem pairs, we show the values of the upstream genes, the downstream ones are very similar (Fig. S3).

If the torsional coupling plays a role in the co-expression, we expect all orientation-dependent features to decay over a distance of around 1000 bases between the genes. Fig. 4 shows that the large majority of both divergent and tandem pairs are indeed located in this range (upper panel), and may thus be transcriptionally coupled. Such a mechanism would increase the expression level as well as the correlation between two genes of a divergent pair, and reduce those of a convergent pair. The fraction of nonzero expression genes (second row) is indeed considerably larger for divergent genes, starting from about 80% for close genes, and decreasing to ∼30% at 3000 bases of distance. Importantly, the smooth decrease seems incompatible with other proposed explanations such as bidirectional promoters, but is fully consistent with the idea that the negative torsion would help opening the promoter with a distance-decreasing strength. In tandem and convergent pairs, the open fraction is indeed lower, but the distance dependence is less clear. The average expression (lower row) presents similar features. We notice that only closeby divergent genes are above the average level of the genome (dashed line). Since these genes are also the most frequent, they represent the overwhelming majority of transcripts in the considered sample (third row). We identified the pairs that exhibit a correlated expression of the two genes in the 24 independent experiments carried on different cell lines (details of the employed criterion are given in the Models section). The correlation is indeed more frequent in closeby divergent genes, where about 20% of the genes are coexpressed, against 5–10% in tandem genes (upper panel, red curve, mind the different scale from the black curve). The curve decreases even faster than the previous ones, with nearly all correlated pairs separated by less than 1000 bases. Altogether, these expression data consistently suggest that the supercoiling-mediated interaction could play an important role in the control of paired gene expression *in vivo*.

## Discussion

We have proposed the first quantitative model of the torsional coupling between adjacent genes, which predicts a particularly strong mutual influence of divergent/convergent pairs, albeit with very different consequences in prokaryotes and eukaryotes. How do these results compare to published experimental data?

Only few quantitative studies followed simultaneously the level of supercoiling and transcription, and they involved mainly prokaryotic genes *in vitro*. In [24], Opel et *al*. followed the expression of a pair of divergent bacterial promoters placed on a plasmid, as a function of the global superhelical level (*i.e.* along a vertical line in the diagram of Fig. 3A). Consistent with our predictions, the expression of the probe gene is triggered at 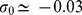 (wrt 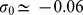 in absence of the second gene), suggesting that the self-reinforcing pair is able to generate a significant local superhelical stress of 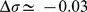 even at a relatively high basal level where the expression of each separate gene is normally low (see Fig. 4 in ref. [24]).

In eukaryotes, the presence of supercoiling is more localized [9] and complicated by the ubiquitous presence of nucleosomes (see below). Still, in the case of divergent promoters, the role of negative supercoiling in the activity of a promoter was demonstrated in a transfected plasmid [25] and recently directly in a human chromosome [26]. To our knowledge, only one study [28] systematically compared the expression level of a pair of genes in the different configurations (divergent, convergent, tandem), in this case two fluorescent genes controlled by the viral promoter CMV, inserted in two genomic sites of the mouse genome (and on both strands in each case). In the divergent and convergent configurations, the results are consistent in both sites and global orientations of the cassette (Fig. S4 A), suggesting that the effect of the chromatin environment or nearby genes is limited. The expression levels of the two genes are also similar in all cases, consistent with the symmetric construction. The divergently oriented genes are systematically expressed around 4 times stronger than the convergent genes (with relatively large deviations), compatible with the diagrams of Fig. 3. For genes placed in tandem, where we predict a lower effect of supercoiling, the results are indeed less clear, with the relative expressions depending on the insertion site and strand, maybe reflecting the influence of the chromatin environment (see Fig. S4 B). Altogether, these results clearly suggest at least a partial role of supercoiling. However, the authors did not mention this possibility [28]. They rather suggested a direct interference between the polymerases transcribing the two genes, although it is difficult to predict even qualitatively how this effect would then depend on the gene orientations. Conversely, the data also illustrate the difficulty of identifying the influence of supercoiling on a single construction in absence of a direct local measurement of σ, where it may be hidden by uncontrolled local features or by more specific regulation mechanisms. Our model, aimed at describing the most systematic effects of supercoiling, is applicable to a wide range of experimental systems with a very limited number of parameters, and may thus help to overcome such problems and distinguish similar effects of superhelicity in independent experiments. More specific features may however lead to deviations from our predictions, which might be taken into account in more involved models, and are discussed in the following paragraphs.

A first simplification is the exponentially-decaying profile of time-averaged superhelical density resulting from transcription. This profile is in agreement with *in vivo* experimental observations in both prokaryotes and eukaryotes [19]–[22]. The similarity between the decay length in both types of organisms was unexpected, considering that nucleosomes cover around 80% of eukaryotic genomes, and are able to store a significant amount of negative supercoiling [5], [21]: their eviction could thus contribute in absorbing the positive stress downstream of a transcribed gene [10], [21], [22], [44]. One possible intuitive explanation is that the total level of supercoiling generated by the elongating polymerase (one turn every 10.5 bases) is anyway considerably larger than the level possibly absorbed by nucleosomes (about one turn every 200 bases): even after their eviction, most of the stress should still be released by topological enzymes. The dynamic rearrangement of nucleosomes around the transcribed region could also complicate considerably the interaction between adjacent genes, and, for time-averaged quantities, result in non-monotonous curves of propagated torsion rather than the simple exponential decay considered here. These interpretation problems reflect the limitations of our time-averaged description of an intrinsically dynamic process, a limitation also present in the available expression data. It may be refined using time-resolved data which only begin to reveal the details of the process [35] (see below).

A second simplification is our nonspecific description of the promoter response to supercoiling. It is well-known that this response depends on the promoter, with non-monotonic expression profiles [24]. For the prokaryotic gene coding for the gyrase enzyme that underwinds DNA, the promoter is even triggered precisely when the DNA is overwound [20]. Such effects deviate from our simple monotonic opening profile. They may be due in part to a sequence-specific contribution to polymerase binding (see details in Models) and subsequent steps of transcription (*e.g.* promoter escape). But another, likely stronger, mechanism is the competition between the opening of the polymerase binding site and structural transitions at distal sites, which involves specific DNA-binding regulatory proteins [14]. Such effects are already present in the *in vitro* model promoters of Fig. 1. For the yeast promoter (C–D), the relatively smooth profile results from the simultaneous opening of a distal site in the employed sequence (around 300 bp ahead of the TSS). If this site is removed and the polymerase binding site alone is included in the calculation, the profile is much sharper and deviates from the data. In contrast, for the bacterial promoter of *pelE* (Fig. 1 A–B), experiments show that transcription occurs only in the presence of the Crp binding protein [11], otherwise the opening of a very unstable distal site absorbs the negative torsion almost entirely, and prevents the opening of the initiation site (S. Reverchon, priv. comm.). Consistently, if we include the full regulatory sequence in the calculation, only the distal site is opened. The expression profile of Fig. 1D was reproduced by including only the polymerase binding site (60 bp), suggesting that Crp binds to the melted distal site and closes its bubble, thereby allowing the formation of the transcription bubble. Interestingly, this kind of subtle mechanical interactions was observed on a widespread scale in bacteria [45], involving a whole class of regulatory proteins which can interact with the polymerase [14], as well as alternate stress-induced structural transitions of the double-helix (B–Z or B–H transition, cruciform formation, G-quadruplex…) [15], [46]. Together, these effects allow a fine-tuning of the supercoiling-dependent response of promoters, and particularly those of stress-response genes involved in regulatory functions [27]. The modification of the physical properties of the double-helix may allow for a rapid re-programming of the expression pattern of the organism, in particular in response of an external stress or during different growth phases [11], [14]. Interestingly, a similar regulation mechanism was observed in the human *MYC* gene, where specific proteins bind to the regulatory sequence FUSE when the latter is melted by negative supercoiling [21]. In eukaryotes, supercoiling could thus also be involved in regulatory mechanisms more complex than considered in the present study, and where nucleosomes are likely to play a crucial role.

An important point to notice is that our model only describes the time-averaged properties of gene expression. How these properties relate to the dynamic, *i.e.* time-dependent mechanisms, is difficult to predict. In particular, an interference between neighbor genes does not necessarily imply that they are actually transcribed simultaneously. If this was the case, *e.g.* for convergent genes, we would then expect the wave of supercoiling of one gene to hit and block the elongating polymerase of the other gene [35], without ever reaching its promoter, an effect that is not included in our model. However, a comparison of the timescales involved in the transcription process suggests that this scenario is likely not the dominant effect. Indeed, measured elongation rates are in the range 20–100 bases/second [47], *i.e.* the elongation phase takes typically less than a minute for usual genes. In contrast, the supercoiling generated by transcription was shown to take around 30–60 minutes to be released by topoisomerases (in human cells) [21]. In most cases, we thus expect that, when one of the gene is transcribed, there is no elongating polymerase on the second gene, and the torsional perturbation can reach its promoter and thus affect its initiation rate for the following ∼30 minutes. For convergent promoters, this rate is reduced, while for divergent genes, if negative supercoiling allows to shortcut the (possibly rate-limiting) requirement of transcription factor recruitment [12], then a transcribed gene could dynamically trigger the expression of its neighbor. However, we also note that many eukaryotic genes are transcribed during short and infrequent events referred to as “transcription bursts” [48], maybe controlled by other factors such as epigenetic modifications or the stochastic recruitment of transcription factors. If these events are rare (separated by more than 30 minutes), then in average the supercoiling generated by the transcription of one gene can be entirely released before the second gene is expressed, and the two genes are torsionally decoupled and we expect no interaction. If this happens for many genes, it might explain the observations of Fig. 4, that only 20% of the close divergent gene pairs are coexpressed. However, such dynamic scenarios remain speculative, when only population-averaged expression data are employed in the analysis. In the future, time-resolved single-cell expression data will allow to properly distinguish the dynamic aspects of the torsion-induced coupling between adjacent genes, and will then justify to consider more involved dynamic models, where the supercoiling should affect not only the initiation rate, but also the elongation of the polymerase in the case where the two genes are elongated simultaneously (in particular convergent genes). Such models will be particularly relevant, since divergent pairs were found to exhibit not only higher expression levels, but also lower expression noise in yeast, which may constitute a characteristic feature of this architecture [41].

In the analysis of RNA-Seq data, we focused on the “torsionally isolated” pairs of genes, where the mutual interaction could be most clearly identified. Only in eukaryotes could we find a sufficiently large number of these genes, and we therefore restricted the analysis to *Drosophila*. It does not mean however that other species are not affected by the interaction, but the small number of these pairs in denser genomes makes it more difficult to test the predictions. This is true in particular for prokaryotes, where the predicted effects are different, but where most promoters are expected to be simultaneously coupled to several other genes, often with different orientations [15]. Even in *Drosophila*, many genes were disregarded because their promoter was within torsional influence of more than a single gene. This situation is probably also frequent in the less compact mammalian genomes, where many genes were found to be densely clustered [39]. In this case, based on the proposed model, we expect a complex simultaneous transcriptional coupling between the (potentially many) genes of the cluster, with each gene affecting directly all promoters in its vicinity, and indirectly the more remote ones. This chain of coupled genes extends until a promoter-less region of ∼3000 bases acts as a “topological insulator” for the transcription. The chain could be very long in the case of dense genomes such as yeast (or prokaryotes), with short-ranged interactions possibly giving rise to collective transitions, as suggested by an analogy to the unidimensional Ising chain. If this transcriptional coupling of adjacent genes plays a functional role, it could thus constitute an eukaryotic equivalent to prokaryotic operons. Although our model theoretically allows to describe such features and numerically compute the result of the collective coupling, we note that the nonlinear interactions between the genes make the behavior strongly dependent on the details of the employed models and computation methods, especially when the number of involved genes increases. With only limited available data, we crucially miss the required precision to embark on the systematic calculation of such effects. We merely note that they would support a functional role for gene clusters, which again differs from the usual idea that closeby genes can only be positively correlated if located in the same chromatin domain. Rather, the orientation-dependence of the torsional coupling could lead to more complex relations between clustered genes.

Importantly, these relations extend not only to coding genes, but also to promoters controlling non-coding transcripts. These promoters have attracted considerable attention recently for their possibly widespread role in transcriptional regulation. Interestingly, while short non-coding RNAs have widely recognized functional roles, that of long ones is less clear, and in particular a subclass of long antisense transcripts [49]. It has been suggested that this regulatory role could be played during their transcription, which would interfere with a coding gene. Again, suggested mechanisms are generally based on direct clashes between the polymerases of the coding and non-coding genes [49], but we expect such clashes to occur for short as well as long RNAs. In contrast, we note that long transcripts are precisely the ones leading to significant amounts of supercoiling. Torsion is thus a potential candidate for a specific mode of action of long non-coding transcripts, which would be particularly strong for antisense ones, and could affect coding promoters even at some kilobases of distance.

## Models

In this paper, we model the time-averaged effect of transcription-induced superhelicity on gene expression. The model is the combination of three ingredients, which are developed in the following paragraphs:

- the spatial distribution of superhelicity generated by a transcribed gene, as described by a mean-field approach
- the supercoiling-dependent opening free energy of a promoter sequence 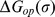

- the thermodynamic model of transcription, which takes 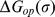 as a key ingredient

### Transcription-induced supercoiling

Consistently with the time-average approximation of gene expression, the distribution of superhelicity σ generated by a transcribed gene is described with a mean-field approach.

The average superhelical density ±σ_a_ at either end of the transcription unit is assumed proportional to the promoter strength *k* and transcript length *l*, consistent with experimental observations [24]. Outside the gene, this stress propagates, while topoisomerase enzymes have a uniform probability 1/b to release the local excess of torsion σ(*x*): 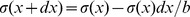. This equation yields an exponentially decaying distribution consistent with experimental observation [19]–[22]:
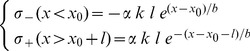
(1)where *x*_0_ and 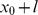 are the beginning and the end of the transcribed unit respectively, and the decay length is given by the topoisomerase efficiency 1/b. This efficiency may depend on topoisomerase concentration (and thus on the organism) as well as on DNA sequence, in particular through sequence-specific transitions of the double-helix [14], [15] (see Discussion), but *in vivo* experiments involving very different organisms and protocols [19]–[22] yielded consistent results in the range of ∼1 kb, which we use as a parameter in the simulations illustrating the model throughout the paper. These simulations (Figs. 2 and 3) involved identical genes of 1000 bases in length and the sequence of the CMV viral promoter (opening profile in Fig. S2) used in the experiments of Fig. S4 [28], [48]. The parameter α was adjusted to generate levels of supercoiling compatible with the experiments [21], for the arbitrary unit of expression used in these simulations (see below).

### Thermodynamic model of DNA

The supercoiling-dependent opening free energy of DNA is estimated from a recent efficient implementation [32] of the Benham model [2], [31], which estimates the opening probabilities of a sequence for given salt and temperature conditions, from the knowledge-based thermodynamic and elastic properties of the double-helix. We checked the robustness of the computation by comparing the melting profiles obtained with the promoter sequence only, or flanked by random sequences of various lengths, with no significant differences. The typical shape of the free energy curve is shown on Fig. 1A, with a transition between and “over” and “undertwisted” states.

For simplicity, the numerical estimations of the torsional coupling included a sigmoidal fit covering the entire crossover: 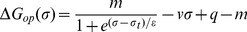
(2)where 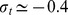 is the sequence-dependent threshold of supercoiling-induced destabilization, 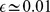 is the width of the transition and *m*, *v*, *q* are adjustable parameters (see the solid lines in Fig. 1 A–C).

For the curves of Fig. 1 A–C, we included the 60 base-pairs sequence ahead of the *pelE* transcription start site (thereby excluding an unstable distal site which competes with the polymerase binding site and is stabilized by the binding of Crp, see Discussion), and the entire 410 bp-sequence ahead of the *CUP1* transcription start site (as used in the experiments), respectively.

Note that for extreme positive torsions (left of the shown curve on Fig. 1A), the thermodynamic model predicts a second destabilization of the double-helix (due to the elastic energy of the double-helical state), which contrasts with the “standard” melting behavior facilitated by negative supercoiling. Assuming that this alternate melting behavior does not occur in the cell in presence of topoisomerases, we did not take it into account in the simulations, and used a monotonous fitted dependence (Eq. 2).

### Transcription under superhelical stress

Following proposed thermodynamic models of transcription [33], [34], the expression level is assumed to be proportional to the initiation probability, as resulting from a chemical equilibrium of bound and unbound states of the transcription machinery. We further assume that the only supercoiling-dependent contribution to the initiation free energy is the opening penalty 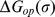 of the promoter, as computed from the thermodynamic model of DNA described in the previous paragraph. The formation of the transcription bubble involves the binding of the polymerase, with an additional contribution 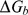, hence a total initiation free energy: 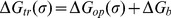
(3)


Throughout the paper, we assume that 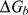 is independent of σ. This hypothesis has strong support for proteins which bind less than 10–15 basepairs, such as many individual transcription factors [34]. Indeed, for the considered supercoiling levels, the twist deformations of the basepairs (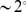/bp) are weaker than the thermal fluctuations at room temperature (standard deviation 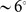/bp) [8] and can be accommodated without substantial energy cost. This statement is valid up to ∼10 basepairs, after which the *correlated* twist modification induced by supercoiling becomes larger than the typical deformations generated by the *uncorrelated* base-pair fluctuations, and may modify significantly the binding properties. This is true in particular for the large RNA Polymerase complex which binds about 30 basepairs of DNA, and where the supercoiling dependence of the initiation free energy may differ from the melting profile. However, this dependence would then be highly specific not only to the supercoiling level but also to the sequence, which would both contribute for instance to the relative position and orientation of the −10 and −35 binding sites of the polymerase [1]. These features may explain the specificity of promoter response to supercoiling [50]. However, since the aim of this paper is to focus on the generic features only, we do not take this dependence into account.

The formation of the transcription bubble is not a purely thermal process, but is facilitated by conformational changes within the RNA polymerase complex. This contribution is difficult to estimate precisely, and probably depends on the type of RNA polymerase. In particular, we expect it to differ between bacterial polymerase which requires no external source of energy to initiate transcription, and eukaryotic RNA PolII which contains an ATP-hydrolysis-dependent helicase subunit [1]. We simply assumed that the equilibrium process takes place at an effective temperature *T*_e_, which defines an energy scale related to the polymerase energetics; this parameter is then adjusted from expression data. For the prokaryotic polymerase of Fig. 1B (see below), we used the value 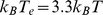 (where *k*_B_ is the Boltzmann constant) best reproducing the experimental curve. Interestingly, we found that the *in vitro* expression data of the eukaryotic promoter *CUP1* (Fig. 1D) are best reproduced by assuming a purely thermal process, 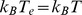. A possible explanation is that these data were obtained in absence of the ATP-consuming transcription factor which ensures the opening of the double helix *in vivo*. In contrast, the *in vivo* data of ref. [28] (Fig. S4) are consistent with a value 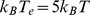, suggesting that the *in vivo* expression is made of two contributions: (i) the thermal opening of underwound promoters and (ii) the assisted opening of relaxed promoters (about 4 times less frequent than the former). Note that because of the relatively large error bars in both experiments, these values are not very precise, but even large modifications would not change the qualitative predictions of the model. For the simulations of Figs. 2 and 3, we chose a value 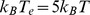, compatible with the eukaryotic *in vivo* expression data of [28] and relatively close to the value found for prokaryotes.

The probability to form a transcript, and hence the average transcription rate *k* of the gene is then given by: 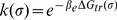
(4)where 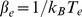 is the Boltzmann factor defining the effective energy scale.

Note that within this framework, the transcription rate fold-change due to supercoiling (as shown on Fig. 3) is independent of 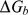, and can thus be computed without detailed knowledge of the binding energetics: 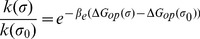
(5)with σ_0_ the basal supercoiling level of the organism.

Together, Eqs. 4 and 1 allow computing the effect of the torsional coupling on the expression of a pair of genes, as a function of their distance, promoter strength and the basal superhelical level (Fig. 3). We integrated the model numerically with an iterative algorithm. Starting from the transcription rate in absence of local supercoiling (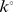) for both genes, the procedure successively adjusts the level of supercoiling (and thus the transcription level) of each promoter until numerical convergence (fixed point). This procedure, as well as all analysis and plotting, were implemented in Python, with the Numpy/Scipy [51] and MatPlotLib [52] libraries.

### Analysis of genome-wide expression data

The RNA-Seq expression data from 24 *D. melanogaster* cell-lines was taken from the November 1st, 2013 release of FlyBase (2013_06 release, library FBlc0000260), and based on communication [43]. They contained the expression levels of ∼16000 genes, including the ∼1500 non-coding genes (detailed information is described on the FlyBase website).

The two genes of a pair were considered as correlated if (i) they were simultaneously expressed in at least 6 of the 24 experiments; (ii) Pearson's correlation coefficient between the 24 pairs of expression levels is larger than 0.5. A modification of these threshold values changed the absolute number of “accepted” pairs, but not significantly the relative number of divergent vs. tandem or convergent correlated pairs.

## Supporting Information

Figure S1 ***In vitro*** **expression profiles of prokaryotic and eukaryotic promoters**. Same data as in Fig. 1 (from [11] and [12] respectively). The expression level is proportional to σ^2^, suggesting that the rate is governed by the promoter melting energy.
(EPS)

FigureS2 **Melting profile of the CMV promoter** [28], [48] **employed in the** **Figures 2** **and** **3** **.**
(EPS)

Figure S3 **Expression properties of downstream genes in** ***Drosophila melanogaster*** **tandem isolated pairs**. The quantities shown in the different rows are identical to those of Fig. 4: (i) Number of gene pairs (black), correlated gene pairs (red); (ii) fraction of expressed genes; (iii) total transcription level; (iv) average transcription level per gene. The expression levels are very comparable to those of the upstream genes, compatible with the weak expected effect of the anisotropy (Fig. 3)
(EPS)

Figure S4 ***In vivo*** **expression data from** [28] **, for genes inserted in tandem in different loci and orientations in the mouse genome.** (A) The expression is always stronger for divergent promoters, consistent with a torsional coupling between the genes. (B) For tandem genes, the expression level depends strongly on the insertion site, consistent with a less systematic effect of supercoiling. On the other hand, the expression level is sometimes (third panel) even larger than in the divergent construction, suggesting other mechanisms not described by our model. This is also the case when a single gene was inserted instead of a pair [28], in which case the comparison is more subtle.
(EPS)
